# Early prophylactic pancreatic stent dislodgement increases the risk of pancreatitis after endoscopic retrograde cholangiopancreatography: A retrospective study

**DOI:** 10.1097/MD.0000000000047131

**Published:** 2026-01-09

**Authors:** Jae Sin Lee, Ik Hyun Jo, Jong Yul Lee, Chang-Nyol Paik, Jae Hyuck Chang

**Affiliations:** a Department of Internal Medicine, Incheon St. Mary’s Hospital, College of Medicine, The Catholic University of Korea, Seoul, Republic of Korea; b Department of Internal Medicine, St. Vincent Hospital, College of Medicine, The Catholic University of Korea, Seoul, Republic of Korea; c Department of Internal Medicine, Bucheon St. Mary’s Hospital, College of Medicine, The Catholic University of Korea, Seoul, Republic of Korea.

**Keywords:** endoscopic retrograde cholangiopancreatography, pancreatitis, risk factors, stents

## Abstract

This study aimed to investigate the association between post-endoscopic retrograde cholangiopancreatography (ERCP) pancreatitis (PEP) and early pancreatic stent dislodgement. A retrospective analysis was conducted on patients who underwent ERCP with prophylactic pancreatic stent placement at 3 academic institutions between January 2010 and December 2022. We evaluated the association between early stent dislodgement (≤24 hours) and PEP as well as the risk factors for early stent dislodgement. A total of 750 patients were analyzed in this study; 75 and 675 patients belonged to the early and non-early dislodgement groups, respectively. Early dislodgement significantly increased the risk of PEP (20.0% vs 7.3%, *P* < .001) and post-ERCP hyperamylasemia (40.0% vs 13.9%, *P* < .001). Early dislodgement was identified as an independent risk factor for PEP (OR, 2.960; 95% CI, 1.552–5.648; *P* = .001). Univariate and multivariate logistic regression analyses of factors related to early stent dislodgement demonstrated that suspected bile duct stone as an indication for ERCP (OR, 2.172; 95% CI, 1.232–3.828; *P* = .007), absence of periampullary diverticulum (OR, 2.206; 95% CI, 1.143–4.259; *P* = .018), and absence of an internal flange of the pancreatic stent (OR, 3.109; 95% CI, 1.689–5.723; *P* < .001) were significantly associated with early stent dislodgment. After propensity score matching adjusted for known risk factors for PEP and early stent dislodgement, early stent dislodgement was a significant risk factor for PEP (OR, 3.571; 95% CI, 1.589–8.028; *P* = .002), and stents without an internal flange (OR, 4.463; 95% CI, 2.214–9.380; *P* < .001) and age < 60 years (OR, 2.134; 95% CI, 1.152–3.955; *P* = .016) were significant risk factors for early dislodgement. In conclusion, early dislodgement of a prophylactic pancreatic stent increases the risk of PEP, and is associated with pancreatic stents without an internal flange.

## 1. Introduction

Endoscopic retrograde cholangiopancreatography (ERCP) is a crucial procedure for the diagnosis and treatment of pancreatic and bile duct disorders. However, ERCP is associated with certain complications including post endoscopic retrograde cholangiopancreatography pancreatitis (PEP). The placement of pancreatic stents has been shown to decrease the incidence of PEP and reduce its severity.^[1-3]^ Therefore, the American Society for Gastrointestinal Endoscopy and the European Society of Gastrointestinal Endoscopy guidelines recommend pancreatic stent placement in patients identified as being at high risk for PEP.^[4,5]^

The protective benefits of a pancreatic stent may be compromised if it is prematurely dislodged from the pancreatic duct (p-duct). However, few studies have investigated the relationship between the early dislodgement of pancreatic stents and PEP. In 1 study examining prophylactic pancreatic stents, 79 patients experienced dislodgement within 24 hours; notably, early dislodgement of stents did not correlate with an increased incidence of PEP, which was contrary to expectations.^[6]^

Therefore, additional investigations are necessary to clarify the association between early stent dislodgement and PEP. Our study investigated the association between early stent dislodgement and PEP, along with the factors contributing to early stent dislodgement.

## 2. Materials and methods

### 2.1. Patients and study design

**Figure 1.**
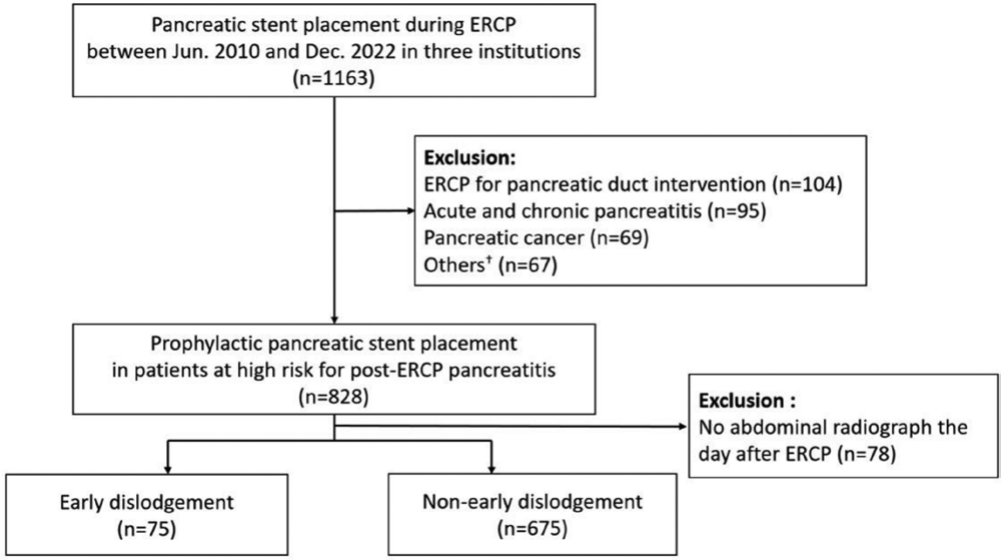
Flowchart illustrating the inclusion and exclusion criteria for the study population. ^†^Diseases involving the pancreas or insufficient data. ERCP = endoscopic retrograde cholangiopancreatography.

This retrospective multicenter study investigated 1163 patients who underwent ERCP with plastic pancreatic stent placement at 3 academic institutions between January 2010 and December 2022 (Fig. 1). Patients were identified from the ERCP registry cohort at each institution. Demographic parameters, ERCP reports, laboratory data, and imaging findings were retrieved from computerized medical records. The inclusion criteria were as follows: ERCP conducted with plastic pancreatic stent placement and age ≥ 18 years. Exclusion criteria: ERCP was conducted for p-duct interventions, such as p-duct stone removal or drainage; patients with pancreatic diseases, including acute pancreatitis, chronic pancreatitis, intraductal papillary mucinous neoplasm, and pancreatic cancer; clinical data was not intact. This study was conducted in accordance with the principles of the Declaration of Helsinki. This study was approved by the Institutional Review Board of Bucheon St. Mary’s Hospital (approval code: HC22RIDI0148). The requirement for informed consent was waived owing to the retrospective and non-interventional nature of this study.

### 2.2. Outcome of interest

The primary outcomes included the association between early pancreatic stent dislodgement and PEP as well as the risk factors for early dislodgement. The secondary outcomes included the association between pancreatic stent characteristics and post-ERCP complications.

### 2.3. Included variables and definitions

Pancreatic stents were categorized based on 3 characteristics: shape, length, and presence or absence of an internal flange. The shapes of pancreatic stents included straight and pigtail, which correspond to the configuration of the duodenal end. The lengths of pancreatic stents were 3, 5, 7, 9, and 10 cm, which were classified as short (3 cm) or long (≥5 cm) stents. The presence or absence of an internal flange determined whether the stent was flanged or unflanged. Post-ERCP complications included hyperamylasemia, acute pancreatitis, and bleeding. Post-ERCP hyperamylasemia was defined as a serum amylase level exceeding 3 times the upper limit of normal following the procedure. PEP was defined as new-onset or exacerbated abdominal pain accompanied by post-ERCP hyperamylasemia. The severity of PEP was classified according to the Cotton criteria: mild pancreatitis (hospitalization for less than 4 days), moderate pancreatitis (hospitalization between 4 and 10 days), and severe pancreatitis (hospitalization exceeding 10 days, development of complications such as necrosis or pseudocyst, or the need for intervention).^[7]^ Post-ERCP bleeding was defined as hematemesis and/or melena, or a decrease in hemoglobin level of >2 g/dL following the procedure. Early stent dislodgement was defined as a case in which the pancreatic stent was dislodged within 1 day. This was confirmed by routine abdominal radiography obtained the day after ERCP. Difficult cannulation was defined as 5 or more cannulation attempts or cannulation time exceeding 10 minutes.

### 2.4. Procedures

ERCP was performed by using a duodenoscope (TJF-260V; Olympus, Tokyo, Japan). A pancreatic stent was inserted along the guidewire in the p-duct during the procedure. p-Duct opacification with contrast was performed when deemed necessary by the operator to evaluate p-duct anatomy. The pancreatic stents used in this study were obtained from a single manufacturer (Cook Endoscopy Inc., Winston-Salem) and were available in either pigtail or straight configuration. The selection of stent shape, length, and diameter and the decision to remove the internal flange were made at the discretion of the operator.

### 2.5. Statistical analysis

The groups with and without early stent dislodgement were compared. Categorical variables were presented as the number of observations, with frequencies reported as percentages. Continuous variables were expressed as mean values ± standard deviation. Pearson’s chi-square test was employed to compare categorical variables, while Fisher’s exact test was used when the expected count in any column was <5. To compare continuous variables, either Student *t* test or Mann–Whitney *U* test was used. Univariate and multivariate analyses employing logistic regression modeling were conducted to identify variables that may be predictive of risk factors for PEP and early dislodgement of the pancreatic stent. The multivariate analysis included variables with *P*-values < .1 or those deemed clinically relevant in univariate analysis. Pancreatic stents with an internal flange are prone to be used in patients at high risk for PEP and are closely related to stent dislodgement. Therefore, propensity score matching was performed to minimize bias that may occur when identifying risk factors for PEP and early dislodgement. Patients were matched 1:1 (internal flange group:no internal flange group) using propensity scores generated using multivariable logistic regression model including known risk factors for PEP and early stent dislodgement like age, sex, difficult cannulation, contrast injection in the p-duct, endoscopic sphincterotomy, endoscopic papillary balloon dilatation, transpancreatic sphincterotomy, and precut sphincterotomy.^[5]^ Nearest neighbor matching was performed with a caliper of 0.1 units of the pooled estimate of the common standard deviation of the logits of the propensity scores. Odds ratios (OR), 95% confidence intervals (CI), and *P*-values were reported to 3 decimal places and rounded off accordingly. Statistical significance was predefined at *P* < .05 for all tests. Statistical analyses were conducted using SAS^®^ software (version 9.4; SAS Institute, Cary).

## 3. Results

### 3.1. Baseline characteristics

A total of 750 patients met the inclusion criteria for analysis. The early stent dislodgement group included 75 (10%) patients, whereas the non-early dislodgement group included 675 (90%) patients. The characteristics of the patients and ERCP procedures are summarized in Table 1. The mean age of the patients was 66.0 years, with the early dislodgement group being significantly younger than the non-early dislodgement group (60.2 years vs 66.6 years, *P* < .001). Male accounted for 50.3% of our patient cohort, with no statistically significant difference in sex ratio between the 2 patient groups. The primary indication for ERCP was suspected bile duct stone (50.0%), which was significantly more prevalent in the early dislodgement group than in the non-early dislodgement group (70.7% vs 47.7%, *P* < .001). A significant proportion of cases (62.3%) involved difficult cannulation, with no statistically significant difference observed between the 2 patient groups. The length of the pancreatic stents varied from 3 to 10 cm, with the majority measuring ≤5 cm (96.1%). The diameter of the stents ranged from 4 to 7 Fr, predominantly 5 Fr (98.3%). Compared to the non-early dislodgement group, the early dislodgement group exhibited significantly higher rates of pancreatic stents with a pigtail end (97.3% vs 89.2%, *P* = .024), shorter length (3 cm; 78.7% vs 67.3%, *P* = .044), and absence of an internal flange (73.3% vs 38.8%, *P* < .001).

**Table 1:** Characteristics of patients with prophylactic pancreatic duct stent placement.

	Total	Early dislodgement	Non-early dislodgement	*P*-value
**Overall**	750	75	675	
Age (yr), mean ± SD	66.0 ± 14.8	60.2 ± 16.1	66.6 ± 14.5	<.001
Male/female	377/373	42/33	335/340	.296
Periampullary diverticulum	204	13	191	.043
ERCP indications				<.001†
Suspicious bile duct stone	375	53	322	
Bile duct stricture	261	13	248	
Ampulla adenoma	69	3	66	
Others*	45	6	39	
ERCP procedures				
Procedure time (min), mean ± SD	31.9 ± 20.0	27.2 ± 15.2	32.4 ± 20.4	.032
Difficult cannulation	467	49	418	.564
Contrast injection in p-duct	403	35	385	.196
EPBD	98	14	84	.130
Endoscopic sphincterotomy	521	57	464	.196
Transpancreatic sphincterotomy	177	13	164	.178
Precut sphincterotomy	148	9	139	.076
Failure of biliary access	83	7	76	.589
Stent characteristics				
Straight/pigtail	75/675	2/73	73/602	.024
Length				.044‡
3 cm	513	59	454	
5 cm	208	13	195	
6 cm	5	0	5	
7 cm	19	2	17	
9 cm	3	0	3	
10 cm	2	1	1	
Diameter				1.000
4 Fr	1	0	1	
5 Fr	737	71	666	
7 Fr	12	4	8	
Internal flange/no internal flange	433/317	20/55	413/262	<.001

EPBD = endoscopic papillary balloon dilatation, ERCP = endoscopic retrograde cholangiopancreatography, SD = standard deviation.

* Bile leak, choledochocele, and bile duct dilatation.

† Suspicious bile duct stone and other indications (bile duct stricture, ampulla adenoma, others) are compared.

‡ 3 cm stents and ≥5cm stents are compared.

### 3.2. Risk factors for post-ERCP pancreatitis

Patients in the early dislodgement group demonstrated a significantly higher incidence of post-ERCP hyperamylasemia (40.0% vs 13.9%, *P* < .001) and PEP (20.0% vs 7.3%, *P* < .001; Table 2). Logistic regression analysis was conducted to identify risk factors for PEP (Table 3). Established risk factors, stent characteristics, and early stent dislodgement were included as variables. Univariate analysis indicated that early stent dislodgement was a significant risk factor for PEP (OR, 3.194; 95% CI, 1.691–6.034; *P* *<* .001). Multivariate analysis indicated that early stent dislodgement was the only significant risk factor for PEP (OR, 2.960; 95% CI, 1.552–5.648; *P* = .001).

**Table 2:** Post-ERCP complications according to early pancreatic stent dislodgement.

Post-ERCP	Total	Early stent dislodgement		*P*-value
complications		Yes (n = 75)	No (n = 675)	
Hyperamylasemia	124	30 (40%)	94 (14%)	<.001
Acute pancreatitis	64	15 (20%)	49 (7%)	<.001
Mild	54	12 (16%)	42 (6%)	
Moderate or severe	10	3 (4%)	7 (1%)	
Bleeding	21	1 (1%)	20 (3%)	.712

ERCP = endoscopic retrograde cholangiopancreatography.

**Table 3:** Univariate and multivariate logistic regression analysis of risk factors for post-ERCP pancreatitis.

Factors	Univariate			Multivariate		
	OR	95% CI	*P*-value	OR	95% CI	*P*-value
Age						
<60	1.551	0.918–2.621	.101	1.480	0.863–2.538	.155
≥60	1					
Sex						
Male	0.862	0.516–1.440	.571			
Female	1					
Early dislodgement						
Yes	3.194	1.691–6.034	<.001	2.960	1.552–5.648	.001
No	1					
Difficult cannulation						
Yes	1.609	0.913–2.834	.100	1.655	0.931–2.941	.086
No	1					
Transpancreatic sphincterotomy						
Yes	1.087	0.601–1.966	.782			
No	1					
Precut sphincterotomy						
Yes	1.040	0.550–1.968	.903			
No	1					
EPBD						
Yes	0.803	0.355–1.815	.598			
No	1					
Stent shape						
Pigtail	0.896	0.393–2.042	.794			
Straight	1					
Stent length						
3 cm	0.872	0.508–1.496	.618			
≥5 cm	1					
Internal flange						
No	1.145	0.684–1.916	.606			
Yes	1					

CI = confidence interval, EPBD = endoscopic papillary balloon dilatation, ERCP = endoscopic retrograde cholangiopancreatograph, OR = odds ratio.

### 3.3. Risk factors for early dislodgement of pancreatic stent

The results of the logistic regression analysis of risk factors for early pancreatic stent dislodgement are shown in Table 4. In univariate analysis, age < 60 years (OR, 2.063; 95% CI, 1.273–3.344; *P* = .003), suspected bile duct stone as an ERCP indication (OR, 2.641; 95% CI, 1.571–4.440; *P* *<* .001), absence of a periampullary diverticulum (OR, 1.882; 95% CI, 1.012–3.502; *P* = .046), pigtail pancreatic stent (OR, 4.426; 95% CI, 1.064–18.415; *P* = .041), pancreatic stent with short length (3 cm; OR, 1.795; 95% CI, 1.010–3.192; *P* = .046), and pancreatic stent without an internal flange (OR, 4.335; 95% CI, 2.540–7.399; *P* *<* .001) were identified as significant risk factors for early stent dislodgement. Multivariate analysis revealed that stents without an internal flange (OR, 3.109; 95% CI, 1.689–5.723; *P* < .001), absence of a periampullary diverticulum (OR, 2.206; 95% CI, 1.143–4.259; *P* = .018), and suspected bile duct stone as an indication for ERCP (OR, 2.172; 95% CI, 1.232–3.828; *P* = .007) were independent risk factors for early stent dislodgement.

**Table 4:** Univariate and multivariate logistic regression analysis of risk factors for early pancreatic stent dislodgement.

Factors	Univariate			Multivariate		
	OR	95% CI	*P*-value	OR	95% CI	*P*-value
Age						
<60	2.063	1.273–3.344	.003	1.565	0.938–2.611	.086
≥60	1					
Sex						
Male	1.292	0.799–2.088	.296			
Female	1					
Indication						
Suspicious bile duct stone	2.641	1.571–4.440	<.001	2.172	1.232–3.828	.007
Others*	1					
Procedure time						
≥30 min	0.751	0.458–1.233	.258			
<30 min	1					
Periampullary diverticulum						
No	1.882	1.012–3.502	.046	2.206	1.143–4.259	.018
Yes	1					
EPBD						
Yes	1.615	0.865–3.014	.132			
No	1					
Transpancreatic sphincterotomy						
Yes	0.653	0.350–1.218	.181			
No	1					
Precut sphincterotomy						
Yes	0.526	0.256–1.081	.081	0.699	0.325–1.504	.359
No	1					
Stent shape						
Pigtail	4.426	1.064–18.415	.041	2.400	0.553–10.420	.243
Straight	1					
Stent length						
3 cm	1.795	1.010–3.192	.046	0.881	0.457–1.698	.705
≥5 cm	1					
Internal flange						
No	4.335	2.540–7.399	<.001	3.109	1.689–5.723	<.001
Yes	1					

EPBD = endoscopic papillary balloon dilatation.

* Bile duct stricture, ampulla adenoma, bile leak, choledochocele, and bile duct dilatation.

### 3.4. Association between pancreatic stent characteristics and post-ERCP complications

The association between pancreatic stent characteristics and post-ERCP complications is shown in Table 5. The parameters included in pancreatic stent characteristics were the shape of the duodenal end (pigtail or straight), stent length (3 cm or ≥5 cm), and presence of an internal flange. No significant association was found between pancreatic stent characteristics and post-ERCP complications.

**Table 5:** Post-ERCP complications according to pancreatic stent characteristics.

Post-ERCP complications	Pigtail	Straight	*P*-value	3 cm	≥5 cm	*P*-value	Flange Yes	Flange No	*P*-value
Hyperamylasemia	111	13	.844	85	39	.941	69	55	.607
Acute pancreatitis	57	7	.794	42	22	.635	35	29	.606
Mild	49	5		35	19		27	27	
Moderate or severe	8	2		7	3		8	2	
Bleeding	19	2	1.000	12	9	.267	16	5	.115

ERCP = endoscopic retrograde cholangiopancreatography.

### 3.5. Analysis after propensity score matching and spontaneous stent dislodgement

**Figure 2.**
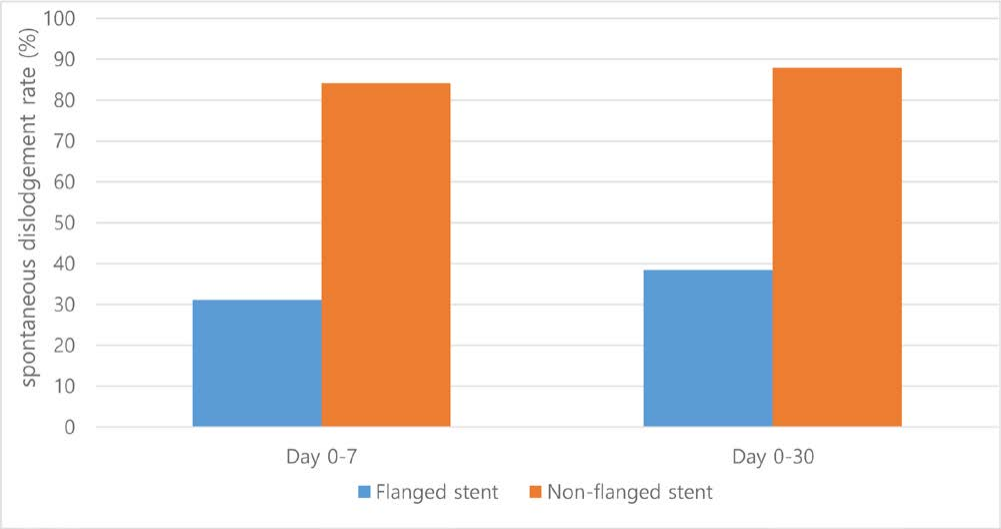
Spontaneous dislodgement rates of pancreatic stents on days 7 and 30 after ERCP. ERCP = endoscopic retrograde cholangiopancreatography.

Table S1, Supplemental Digital Content, https://links.lww.com/MD/R120 shows the patient characteristics of the flanged and non-flanged groups before and after propensity score matching, adjusted for known risk factors for PEP and early stent dislodgement including age, sex, difficult cannulation, contrast injection in the p-duct, endoscopic sphincterotomy, endoscopic papillary balloon dilatation, transpancreatic sphincterotomy, and precut sphincterotomy. The differences in many PEP risk factors between the 2 groups disappeared after propensity matching. Logistic regression analysis was conducted to identify risk factors for PEP and early dislodgement in the matched cohort (Tables 6 and 7). Multivariate analysis indicated that early stent dislodgement was a significant risk factor for PEP (OR, 3.571; 95% CI, 1.589–8.028; *P* =* *.002), and stents without an internal flange (OR, 4.463; 95% CI, 2.214–9.380; *P* < .001) and age < 60 years (OR, 2.134; 95% CI, 1.152–3.955; *P* = .016) were significant risk factors for early dislodgement. Figure 2 shows the spontaneous dislodgement rates for flanged and non-flanged pancreatic stents on days 7 and 30 after ERCP, respectively. A total of 283 patients whose pancreatic stent dislodgement time could be ascertained were included in the figure, with 151 flanged stents and 132 non-flanged stents. The spontaneous dislodgement rates on days 7 and 30 after ERCP were significantly higher in the non-flanged group than in the flanged group (84.1 vs 31.1%; 87.9% vs 38.4%; *P* < .001, respectively).

**Table 6:** Univariate and multivariate logistic regression analysis of risk factors for post-ERCP pancreatitis after propensity score matching.

Factors	Univariate			Multivariate		
	OR	95% CI	*P*-value	OR	95% CI	*P*-value
Age						
<60	1.206	0.570–2.552	.625			
≥60						
Sex						
Male	0.710	0.352–1.432	.339			
Female						
Early dislodgement						
Yes	3.671	1.643–8.200	<.001	3.571	1.589–8.028	.002
No						
Difficult cannulation						
Yes	2.283	1.009–5.165	.048	2.215	0.972–5.049	.059
No						
Endoscopic sphincterotomy						
Yes	1.078	0.455–2.557	.865			
No						
Transpancreatic sphincterotomy						
Yes	0.695	0.160–3.025	.628			
No						
Precut sphincterotomy						
Yes	0.663	0.226–1.942	.453			
No						
EPBD						
Yes	1.210	0.481–3.042	.686			
No						
Stent shape						
Pigtail	1.684	0.219–12.942	.617			
Straight						
Stent length						
3 cm	1.160	0.465–2.893	.751			
≥5 cm						
Internal flange						
No	1.291	0.639–2.611	.476			
Yes						

CI = confidence interval, EPBD = endoscopic papillary balloon dilatation, ERCP = endoscopic retrograde cholangiopancreatography, OR = odds ratio.

**Table 7:** Univariate and multivariate logistic regression analysis of risk factors for early pancreatic stent dislodgement after propensity score matching.

Factors	Univariate			Multivariate		
	OR	95% CI	*P*-value	OR	95% CI	*P*-value
Age						
<60	1.992	1.101–3.607	.023	2.134	1.152–3.955	.016
≥60						
Sex						
Male	1.360	0.752–2.459	.310			
Female						
ERCP Indication						
Suspicious bile duct stone	2.374	1.287–4.379	.006	1.648	0.864–3.142	.129
Others*						
Procedure time						
≥30 min	0.602	0.326–1.110	.104			
<30 min						
Periampullary diverticulum						
No	1.586	0.769–3.272	.212			
Yes						
EPBD						
Yes	1.586	0.770–3.266	.211			
No						
Endoscopic sphincterotomy						
Yes	1.040	0.513–2.110	.913			
No						
Transpancreatic sphincterotomy						
Yes	1.583	0.625–4.006	.333			
No						
Precut sphincterotomy						
Yes	0.510	0.196–1.330	.169			
No						
Stent shape						
Pigtail	1.266	0.286–5.600	.755			
Straight						
Stent length						
3 cm	1.401	0.634–3.093	.404			
≥5 cm						
Internal flange						
No	4.943	2.412–10.129	<.001	4.463	2.124–9.380	<.001
Yes						

CI = confidence interval, EPBD = endoscopic papillary balloon dilatation, ERCP = endoscopic retrograde cholangiopancreatography, OR = odds ratio.

* Bile duct stricture, ampulla adenoma, bile leak, choledochocele, and bile duct dilatation.

## 4. Discussion

The present study demonstrated that early dislodgement of prophylactic pancreatic stents increased the risk of PEP. Early dislodgement of the pancreatic stent is associated with pancreatic stents without an internal flange.

It is well established that placement of a pancreatic stent is effective in preventing PEP. Consequently, ERCP guidelines recommend the placement of a pancreatic stent in populations at high risk of pancreatitis.^[4,5]^ Nevertheless, the optimal duration for maintaining the pancreatic stent to prevent PEP remains unclear. The incidence of early pancreatic stent dislodgement has been reported to range from 13% to 50% for stents without an internal flange.^[8,9]^ Prevention of pancreatitis may be compromised if the placed pancreatic stent is inadvertently dislodged prematurely. However, studies on this topic are limited. Moffatt et al conducted a retrospective study to determine the effect of early dislodgement of a pancreatic stent on PEP. The study demonstrated that dislodgement of a pancreatic stent within 72 hours was not associated with an increased risk of PEP or a more severe course of PEP.^[8]^ Conversely, when the pancreatic stent was removed immediately at the conclusion of ERCP, the incidence of PEP increased.^[10,11]^ In patients anticipated to experience severe pancreatitis, in whom a pancreatic stent had not been placed or where early dislodgement occurred, salvage placement of a pancreatic stent following ERCP was reported to facilitate prompt PEP resolution.^[12]^ Our study revealed that early stent dislodgement is significantly associated with post-ERCP hyperamylasemia and pancreatitis. To the best of our knowledge, this is the first report to indicate that early pancreatic stent dislodgement (≤24 hour) mitigates its preventive effect on PEP. Given that typical PEP usually begins within a few hours after ERCP and becomes apparent within 1 day,^[13-15]^ we established the criterion for early dislodgement as occurring within 1 day and identified an association between early stent dislodgement and PEP. Moffatt et al employed a threshold of 72 hours for early pancreatic stent dislodgement and reported that early stent dislodgement was not associated with moderate or severe PEP.^[8]^

Our study identified factors associated with the early dislodgement of p-duct stents. The independent risk factors for early dislodgement included suspected bile duct stone as an indication for ERCP, absence of a periampullary diverticulum, and absence of an internal flange on the stent. Among these, the absence of an internal flange was the only independent risk factor after propensity score matching. The internal flange was specifically designed to prevent stent dislodgement. However, the guidelines recommend the use of a prophylactic pancreatic stent without an internal flange, as this approach minimizes the need for additional endoscopic procedures by promoting spontaneous dislodgement.^[4,5]^ Considering the disadvantages associated with an internal flange, including the requirement for follow-up endoscopy and the potential risk of p-duct injury resulting from the pancreatic stent,^[16-18]^ it is advisable to use a stent without an internal flange in patients undergoing ERCP.^[19-21]^ However, based on our study, the use of a stent with an internal flange should be considered in patients at high risk of early stent dislodgement.

Pancreatic stents without an internal flange are closely associated with stent dislodgement, as shown in Figure 2. Moreover, pancreatic stents with an internal flange may have the potential to be used in patients at high risk for PEP. Therefore, propensity score matching was performed to minimize bias that may occur when identifying the risk factors for PEP and early dislodgement in the total cohort.

Various pancreatic stents with differing characteristics, including shape, diameter, length, and presence or absence of a flange, are utilized in clinical practice. The association between stent shape, length, and internal flange with PEP risk has not been found or clearly established in previous studies.^[9,22-25]^ In the present study, no association was observed between the characteristics of pancreatic stents and the incidence of PEP. This finding suggests that the properties of each stent were not definitively associated with PEP. Nonetheless, our study revealed an association between PEP and early dislodgement as well as between early dislodgement and internal flanges. This finding implies that although the internal flange may not be a sufficiently significant factor influencing PEP, it is related to PEP in cases of early dislodgement. Concerning stent diameter, a network meta-analysis comparing 5 and 3 Fr pancreatic stents demonstrated that the 5 Fr stent was superior to the 3 Fr stent in preventing PEP in high-risk patients.^[26]^ Furthermore, Zolotarevsky et al reported that placement of the 5 Fr stent was easier and faster than that of the 3 Fr stents, with no observed difference in spontaneous passage.^[27]^ However, in our study, the majority of stents were 5 Fr, which precluded a comparison of p-duct stent diameters.

This study has several limitations. First, its retrospective nature may introduce variability in the availability of relevant data. However, all 3 institutions adhered to identical procedural protocols and post-procedure monitoring standards. Second, rectal nonsteroidal anti-inflammatory drugs were not administered either before or after ERCP in this study. Research indicates that rectal nonsteroidal anti-inflammatory drugs can effectively prevent PEP, suggesting that their use may be beneficial.^[28,29]^ A recent randomized controlled trial demonstrated that indomethacin alone was less effective than indomethacin in combination with prophylactic pancreatic stent placement in high-risk patients.^[30]^

In conclusion, early stent dislodgement was a risk factor for PEP. The use of a stent with a flange should be considered in patients at high risk of early stent dislodgement of the pancreatic stent.

## Acknowledgments

We would like to thank Editage (www.editage.co.kr) for editing and reviewing this manuscript for the English language. We are grateful to the Research Factory (www.rfactory.kr) for their assistance with the statistical analysis.

## Author contributions

**Conceptualization:** Jae Hyuck Chang.

**Data curation:** Jae Sin Lee, Ik Hyun Jo, Jong Yul Lee, Chang-Nyol Paik, Jae Hyuck Chang.

**Formal analysis:** Jae Sin Lee.

**Investigation:** Jae Sin Lee, Ik Hyun Jo, Jae Hyuck Chang.

**Methodology:** Jae Sin Lee, Jae Hyuck Chang.

**Validation:** Jae Sin Lee, Jae Hyuck Chang.

**Writing – original draft:** Jae Sin Lee.

**Writing – review & editing:** Jae Hyuck Chang.

## Supplementary Material



## References

[1] Phillip V Pukitis A Epstein A. Pancreatic stenting to prevent post-ERCP pancreatitis: a randomized multicenter trial. Endosc Int Open. 2019;7:E860–8. 31281872 10.1055/a-0886-6384 PMC6609234

[2] Kawaguchi Y Ogawa M Omata F Ito H Shimosegawa T Mine T. Randomized controlled trial of pancreatic stenting to prevent pancreatitis after endoscopic retrograde cholangiopancreatography. World J Gastroenterol. 2012;18:1635–41. 22529693 10.3748/wjg.v18.i14.1635 PMC3325530

[3] Fazel A Quadri A Catalano MF Meyerson SM Geenen JE. Does a pancreatic duct stent prevent post-ERCP pancreatitis? A prospective randomized study. Gastrointest Endosc. 2003;57:291–4. 12612504 10.1067/mge.2003.124

[4] Buxbaum JL Freeman M Amateau SK. American Society for Gastrointestinal Endoscopy guideline on post-ERCP pancreatitis prevention strategies: summary and recommendations. Gastrointest Endosc. 2023;97:153–62. 36517310 10.1016/j.gie.2022.10.005

[5] Dumonceau JM Kapral C Aabakken L. ERCP-related adverse events: European Society of Gastrointestinal Endoscopy (ESGE) guideline. Endoscopy. 2020;52:127–49. 31863440 10.1055/a-1075-4080

[6] Chahal P Tarnasky PR Petersen BT. Short 5Fr vs long 3Fr pancreatic stents in patients at risk for post-endoscopic retrograde cholangiopancreatography pancreatitis. Clin Gastroenterol Hepatol. 2009;7:834–9. 19447196 10.1016/j.cgh.2009.05.002

[7] Cotton PB Lehman G Vennes J. Endoscopic sphincterotomy complications and their management: an attempt at consensus. Gastrointest Endosc. 1991;37:383–93. 2070995 10.1016/s0016-5107(91)70740-2

[8] Moffatt DC Pradermchai K Avula H Sherman S Fogel EL Lehman GA. Moderate and severe postendoscopic retrograde cholangiopancreatography pancreatitis despite prophylactic pancreatic stent placement: the effect of early prophylactic pancreatic stent dislodgement. Can J Gastroenterol. 2011;25:215–9. 21523263 10.1155/2011/678540 PMC3088697

[9] Fujisawa T Kagawa K Ochiai K. Prophylactic efficacy of 3- or 5-cm pancreatic stents for preventing post-ERCP pancreatitis: a prospective, randomized trial. J Clin Gastroenterol. 2016;50:e30–4. 26280707 10.1097/MCG.0000000000000397

[10] Cha SW Leung WD Lehman GA. Does leaving a main pancreatic duct stent in place reduce the incidence of precut biliary sphincterotomy-associated pancreatitis? A randomized, prospective study. Gastrointest Endosc. 2013;77:209–16. 23084272 10.1016/j.gie.2012.08.022

[11] Conigliaro R Manta R Bertani H. Pancreatic duct stenting for the duration of ERCP only does not prevent pancreatitis after accidental pancreatic duct cannulation: a prospective randomized trial. Surg Endosc. 2013;27:569–74. 22926890 10.1007/s00464-012-2487-x

[12] Kerdsirichairat T Attam R Arain M Bakman Y Radosevich D Freeman M. Urgent ERCP with pancreatic stent placement or replacement for salvage of post-ERCP pancreatitis. Endoscopy. 2014;46:1085–94. 25216326 10.1055/s-0034-1377750

[13] Gottlieb K Sherman S Pezzi J Esber E Lehman GA. Early recognition of post-ERCP pancreatitis by clinical assessment and serum pancreatic enzymes. Am J Gastroenterol. 1996;91:1553–7. 8759660

[14] Thomas PR Sengupta S. Prediction of pancreatitis following endoscopic retrograde cholangiopancreatography by the 4-h post procedure amylase level. J Gastroenterol Hepatol. 2001;16:923–6. 11555108 10.1046/j.1440-1746.2001.02547.x

[15] Testoni PA Bagnolo F Caporuscio S Lella F. Serum amylase measured four hours after endoscopic sphincterotomy is a reliable predictor of postprocedure pancreatitis. Am J Gastroenterol. 1999;94:1235–41. 10235200 10.1111/j.1572-0241.1999.01072.x

[16] Smith MT Sherman S Ikenberry SO Hawes RH Lehman GA. Alterations in pancreatic ductal morphology following polyethylene pancreatic stent therapy. Gastrointest Endosc. 1996;44:268–75. 8885345 10.1016/s0016-5107(96)70163-3

[17] Kozarek RA. Pancreatic stents can induce ductal changes consistent with chronic pancreatitis. Gastrointest Endosc. 1990;36:93–5. 2335298 10.1016/s0016-5107(90)70958-3

[18] Sherman S Hawes RH Savides TJ. Stent-induced pancreatic ductal and parenchymal changes: correlation of endoscopic ultrasound with ERCP. Gastrointest Endosc. 1996;44:276–82. 8885346 10.1016/s0016-5107(96)70164-5

[19] Moffatt DC Coté GA Fogel EL. Acute pancreatitis after removal of retained prophylactic pancreatic stents. Gastrointest Endosc. 2011;73:980–6. 21521566 10.1016/j.gie.2011.01.012

[20] Rashdan A Fogel EL McHenry L Jr Sherman S Temkit M Lehman GA. Improved stent characteristics for prophylaxis of post-ERCP pancreatitis. Clin Gastroenterol Hepatol. 2004;2:322–9. 15067627 10.1016/s1542-3565(04)00062-x

[21] Freeman ML. Role of pancreatic stents in prevention of post-ERCP pancreatitis. Jop. 2004;5:322–7. 15365198

[22] Choudhary A Bechtold ML Arif M. Pancreatic stents for prophylaxis against post-ERCP pancreatitis: a meta-analysis and systematic review. Gastrointest Endosc. 2011;73:275–82. 21295641 10.1016/j.gie.2010.10.039

[23] Iqbal S Shah S Dhar V Stavropoulos SN Stevens PD. Is there any difference in outcomes between long pigtail and short flanged prophylactic pancreatic duct stents? Dig Dis Sci. 2011;56:260–5. 20464492 10.1007/s10620-010-1262-x

[24] Minami K Iwasaki E Kawasaki S, . A long (7 cm) prophylactic pancreatic stent decreases incidence of post-endoscopic papillectomy pancreatitis: a retrospective study. Endosc Int Open. 2019;7:E1663–70. 31788550 10.1055/a-1010-5581 PMC6877413

[25] Elmunzer BJ Zhang J Coté GA, . Technical factors associated with the benefit of prophylactic pancreatic stent placement during high-risk endoscopic retrograde cholangiopancreatography: a secondary analysis of the SVI trial data set. Am J Gastroenterol. 2025;120:811–5. 39207308 10.14309/ajg.0000000000003052 PMC11865352

[26] Afghani E Akshintala VS Khashab MA, . 5-Fr vs. 3-Fr pancreatic stents for the prevention of post-ERCP pancreatitis in high-risk patients: a systematic review and network meta-analysis. Endoscopy. 2014;46:573–80. 24830399 10.1055/s-0034-1365701

[27] Zolotarevsky E Fehmi SM Anderson MA, . Prophylactic 5-Fr pancreatic duct stents are superior to 3-Fr stents: a randomized controlled trial. Endoscopy. 2011;43:325–30. 21455872 10.1055/s-0030-1256305 PMC3514442

[28] Elmunzer BJ Scheiman JM Lehman GA. A randomized trial of rectal indomethacin to prevent post-ERCP pancreatitis. N Engl J Med. 2012;366:1414–22. 22494121 10.1056/NEJMoa1111103 PMC3339271

[29] Sperna Weiland CJ Engels MML Poen AC. Increased use of prophylactic measures in preventing post-endoscopic retrograde cholangiopancreatography pancreatitis. Dig Dis Sci. 2021;66:4457–66. 33630216 10.1007/s10620-020-06796-0 PMC8589790

[30] Elmunzer BJ Foster LD Serrano J. Indomethacin with or without prophylactic pancreatic stent placement to prevent pancreatitis after ERCP: a randomised non-inferiority trial. Lancet. 2024;403:450–8. 38219767 10.1016/S0140-6736(23)02356-5 PMC10872215

