# Supplementary material for: Early prophylactic pancreatic stent dislodgement increases the risk of pancreatitis after endoscopic retrograde cholangiopancreatography: A retrospective study
Source: Medicine (Baltimore). 2026 Jan 9;105(2):e47131. doi: 10.1097/MD.0000000000047131 (PMC12795032; doi:10.1097/MD.0000000000047131)
Supplement: Supplementary file 1 [file medi-105-e47131-s001.docx]

**Supplementary Table 1.** Characteristics of patients according to internal flange before and after propensity score matching

|  | *Before matched (n=750)* | | | *After matched (n=446)* | | |
| --- | --- | --- | --- | --- | --- | --- |
|  | No internal flange (%) | Internal flange (%) | *P*-value | No internal flange (%) | Internal flange (%) | *P*-value |
| **Overall** | 317 | 433 |  | 223 | 223 |  |
| Age (y), mean ± SD | 63.4±15.4 | 67.8±14.1 | <0.001 | 66.2 ± 14.3 | 66.8 ± 14.7 | 0.645 |
| Male/Female | 158/159 | 219/214 | 0.842 | 118/105 | 127/96 | 0.392 |
| Early dislodgement | 55/262 | 20/413 | <0.001 | 42 (18.8) | 10 (4.5) | < 0.001 |
| Periampullary diverticulum | 91 | 113 | 0.428 | 67 (30.0) | 51 (22.9) | 0.086 |
| ERCP indications |  |  | < 0.001^*^ |  |  | < 0.001^*^ |
| Suspicious bile duct stone | 214 | 161 |  | 141 (63.2) | 77 (34.5) |  |
| Bile duct stricture | 67 | 194 |  | 56 (25.1) | 124 (55.6) |  |
| Ampulla adenoma | 9 | 60 |  | 18 (8.1) | 11 (4.9) |  |
| Others^†^ | 27 | 18 |  | 8 (3.6) | 11 (4.9) |  |
| **ERCP procedures** |  |  |  |  |  |  |
| Procedure time (min), mean  ± SD | 27.9±16.9 | 34.7±21.5 | <0.001 | 28.9 ± 17.4 | 35.1 ± 22.0 | 0.001 |
| Difficult cannulation | 189 (59.6%) | 278 (64.2%) | 0.201 | 130 (58.3) | 138 (61.9) | 0.439 |
| Contrast injection in p-duct | 120 (37.8%) | 283 (65.4%) | <0.001 | 99 (44.4) | 105 (47.1) | 0.568 |
| EPBD | 49 (15.5%) | 49 (11.3%) | 0.097 | 34 (15.2) | 34 (15.2) | >0.999 |
| Endoscopic sphincterotomy | 266 (83.9%) | 255 (58.9%) | <0.001 | 172 (77.1) | 177 (79.4) | 0.566 |
| Transpancreatic sphincterotomy | 16 (5.0%) | 161 (37.2%) | <0.001 | 16 (7.2) | 20 (9.0) | 0.487 |
| Precut sphincterotomy | 37 (11.7%) | 111 (25.6%) | <0.001 | 35 (15.7) | 38 (17.0) | 0.701 |
| Failure of biliary access | 10 (3.2%) | 73 (16.9%) | <0.001 | 10 (4.5) | 13 (5.9) | 0.499 |
| **Stent characteristics** |  |  |  |  |  |  |
| Straight / Pigtail | 6/311 | 69/364 | <0.001 | 3/220 | 18/205 | <0.001 |
| Length |  |  | <0.001^**^ |  |  | 0.013^**^ |
| 3 cm | 275 (86.8%) | 238 (55.0%) |  | 191 (85.7) | 167 (74.9) |  |
| 5 cm | 34 (10.7%) | 174 (40.2%) |  | 24 (10.8) | 48 (21.5) |  |
| 6 cm | 0 (0%) | 5 (1.2%) |  | 0 (0.0) | 0 (0.0) |  |
| 7 cm | 7 (2.2%) | 12 (2.8%) |  | 7 (3.1) | 6 (2.7) |  |
| 9 cm | 0 (0%) | 3 (0.7%) |  | 0 (0.0) | 2 (0.9) |  |
| 10 cm | 1 (0.3%) | 1 (0.2%) |  | 1 (0.4) | 0 (0.0) |  |
| Diameter |  |  | 0.423 |  |  | >0.999 |
| 4 Fr | 1 | 0 |  | 0 (0.0) | 0 (0.0) |  |
| 5 Fr | 311 | 426 |  | 219 (98.2) | 219 (98.2) |  |
| 7 Fr | 5 | 7 |  | 4 (1.8) | 4 (1.8) |  |

^†^Bile leak, choledochocele, and bile duct dilatation.

^*^Suspicious bile duct stone and other indications (bile duct stricture, ampulla adenoma, and others) are compared.

^**^3 cm stents and ≥ 5cm stents are compared.

SD, standard deviation; EPBD, endoscopic papillary balloon dilatation
